# Responses of roots and rhizosphere of female papaya to the exogenous application of GA_3_

**DOI:** 10.1186/s12870-022-04025-6

**Published:** 2023-01-16

**Authors:** Yongmei Zhou, Ziqin Pang, Haifeng Jia, Zhaonian Yuan, Ray Ming

**Affiliations:** 1 Center for Genomics and Biotechnology, Fujian Provincial Key Laboratory of Haixia Applied Plant Systems Biology, Fujian Agriculture and Forestry University, Fuzhou, 350002 Fujian China; 2 Key Laboratory of Sugarcane Biology and Genetic Breeding, Ministry of Agriculture, Fujian Agriculture and Forestry University, Fuzhou, 350002 China

**Keywords:** Papaya, Root, rhizosphere, Metabolome, Microbiome, Transcriptome

## Abstract

Exogenous GAs have an indeterminate effect on root development. Our current study used female papaya to reveal how the roots and rhizosphere respond to the exogenous application of GA_3_ by investigating the transcriptome profile in roots, metabolic profile and microbial community in both roots and rhizosphere of GA_3_-treated and control female papaya. The results demonstrated that exogenous GA_3_ treatment enhanced female papaya lateral root development, which gave plants physical advantages of water and nutrient uptake. In addition, it was likely that GA_3_ spraying in papaya shoot apices increased the level of auxin, which was transported to roots by CpPIN1, where auxin upregulated *CpLBD16* and repressed *CpBP* to promote the lateral root initiation and development. In papaya roots, corresponding transporters (*CpTMT3*, *CpNRT1:2*, *CpPHT1;4*, *CpINT2*, *CpCOPT2*, *CpABCB11*, *CpNIP4;1*) were upregulated and excretion transporters were downregulated such as *CpNAXT1* for water and nutrients uptake with exogenous GA_3_ application. Moreover, in GA_3_-treated papaya roots, *CpALS3* and *CpMYB62* were downregulated, indicating a stronger abiotic resistance to aluminum toxic and phosphate starvation. On the other hand, BRs and JAs, which involve in defense responses, were enriched in the roots and rhizosphere of GA_3_-treated papayas. The upregulation of the two hormones might result in the reduction of pathogens in roots and rhizosphere such as *Colletotrichum* and *Verticillium*. GA_3_-treated female papaya increased the abundance of beneficial bacteria species including *Mycobacterium*, *Mitsuaria*, and *Actinophytocola*, but decreased that of the genera *Candidatus* and *Bryobacter* for that it required less nitrate. Overall, the roots and rhizosphere of female papaya positively respond to exogenous application of GA_3_ to promote development and stress tolerance. Treatment of female papaya with GA3 might result in the promotion of lateral root formation and development by upregulating *CpLBD16* and downregulating *CpBP*. GA_3_-treated papaya roots exhibited feedback control of brassinolide and jasmonate signaling in root development and defense. These findings revealed complex response to a growth hormone treatment in papaya roots and rhizosphere and will lead to investigations on the impact of other plant hormones on belowground development in papaya.

**Supplementary Information:**

The online version contains supplementary material available at 10.1186/s12870-022-04025-6.

## Introduction

Gibberellins (GAs) are tetracyclic diterpenoid plant hormones that has a chemical structure of gibberellane skeleton. More than 136 GAs have been currently identified from plants, fungi and bacteria, but a few are functionally active like GA_1_, GA_3_, GA_4_, GA_5_, GA_6_ and GA_7_ [1, 2]. The plant growth regulators influence a range of developmental processes in higher plants including stem elongation, seed germination, flowering, sex expression, enzyme induction and leaf and fruit senescence [3, 4]. Therefore, they were widely used in enhancing the productivity of commercial crops [5]. For example, famers use gibberellins to spray on the grapevines to decrease the compactness of clusters but increase berry size to make the clusters of yaghooti grape more popular among consumers [6]. Gibberellins were sprayed on the dwarf peas to reverse their genetic defects [7].

Papaya (*Carica papaya* L.) is a trioecious species, sex types of which were determined by a pair of nascent sex chromosomes, XX for females, XY for males, and XYh for hermaphrodites [8]. Females and hermaphrodites can bear fruits which are rich in vitamin C, carotene and protease. A previous study demonstrated that exogenous application of GA_3_ on female papaya increased its height, peduncle length and inflorescence branch number [9]. While plant phenotype is a consequence of complex interactions between plants and environment, aboveground, the vegetative growth and yield were improved by GA_3_ application [9, 10]. Belowground, however, the responses of roots and rhizosphere (the soil adjacent to the roots) of papaya to the exogenous application have not been studied. These aspects on other plants have also received relatively little attention [11].

Root is an important vegetative organ in contact between plants and soil environment. It holds the stem and participates in water and nutrient uptake, which directly affect plant growth, development and yield [12]. Rhizosphere is the region few millimeters extended from a root system. It is a dynamic region containing a tremendous amount of rhizodeposits, governed by complex interactions between plants and the microorganisms that are in close association with roots [13]. In rhizosphere, the plants and microorganisms exhibit a symbiotic relationship by meeting each other's nutrient requirements [14]. The microorganisms convert organic matter into inorganic matter and provide effective nutrients for plants. Additionally, microorganisms can also secrete vitamin and hormone to promote plant growth [15].

A better understanding of the interactions between plants and environment is necessary to manipulate them to improve plant productivity [13]. In the current work, female papaya (*Carica papaya* L. cv. Zhonghuang) was used as an object to reveal how the roots and rhizosphere respond to the exogenous application of GA_3_ by investigating the transcriptome profile in roots, metabolic profile and microbial community in both roots and rhizosphere of GA_3_-treated and control female papaya.

## Results

### Effects of GA_3_ on morphology and transcriptome profiles in the roots of female papaya

**Fig. 1:**
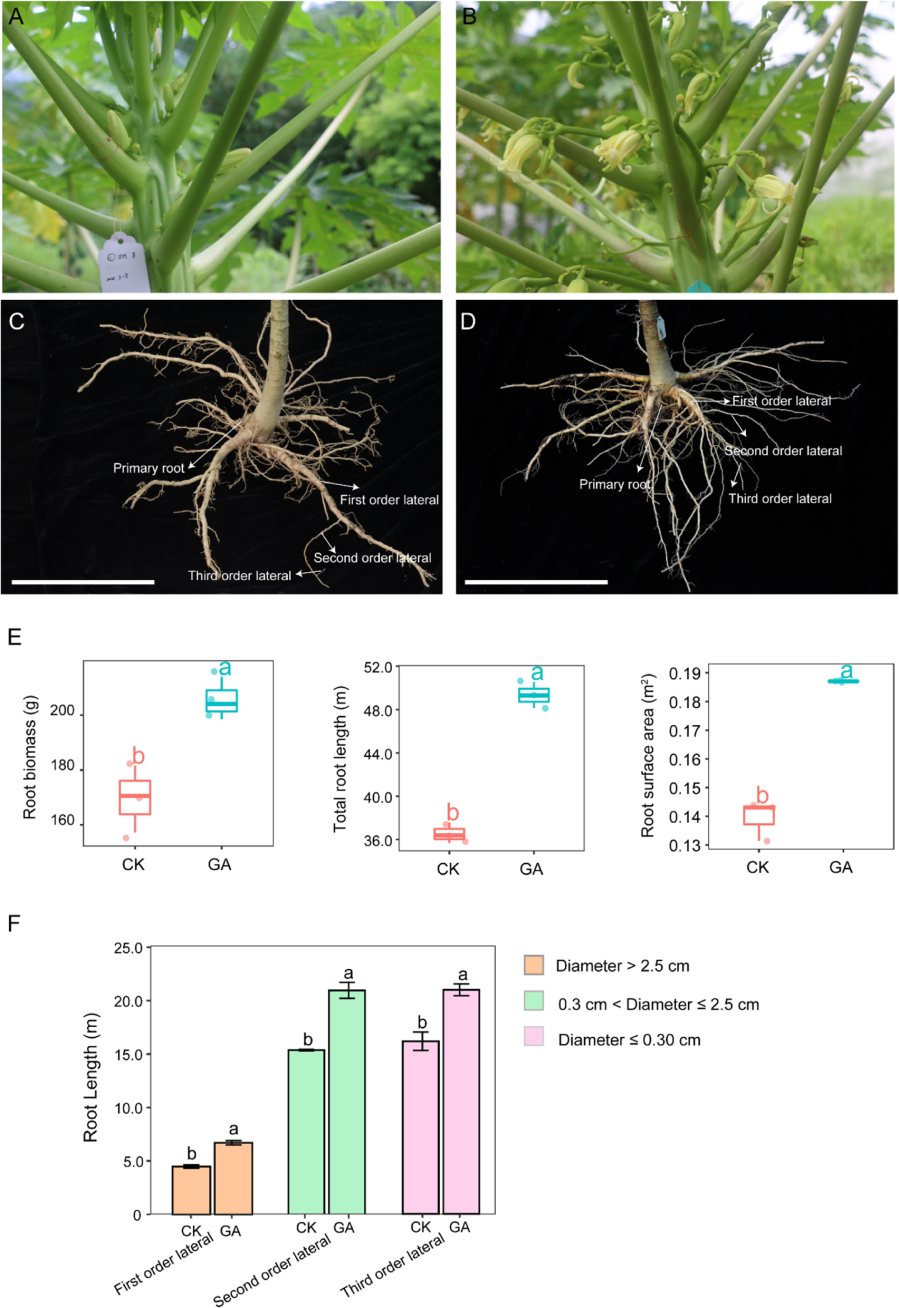
**A**-**B** Physical appearance of inflorescence of CK (**A**) and GA_3_-treated papaya (*Carica papaya* L. cv. Zhonghuang) (**B**). **C**, **D** Root system of CK (**C**) and GA_3_-treated papaya (**D**). Bar=20cm in (**C**) and (**D**). **E** The root biomass, total length and root surface area of CK and GA_3_-treated papaya. **F** The total first order, second order and third order lateral roots of CK and GA3-treated papaya

Compared to the control (CK) group, application of 150 µM GA_3_ on the shoot apex of female papaya resulted in a significantly increase in the peduncle length and flower number (Fig. 1A-B). Notably, GA_3_-treated papaya showed a stronger root system in comparison with control papaya (Fig. 1C-D). GA_3_ application on papaya not only significantly increased the biomass, total length and surface area of roots, but also enhanced lateral root development (Fig. 1E-F).

**Fig. 2:**
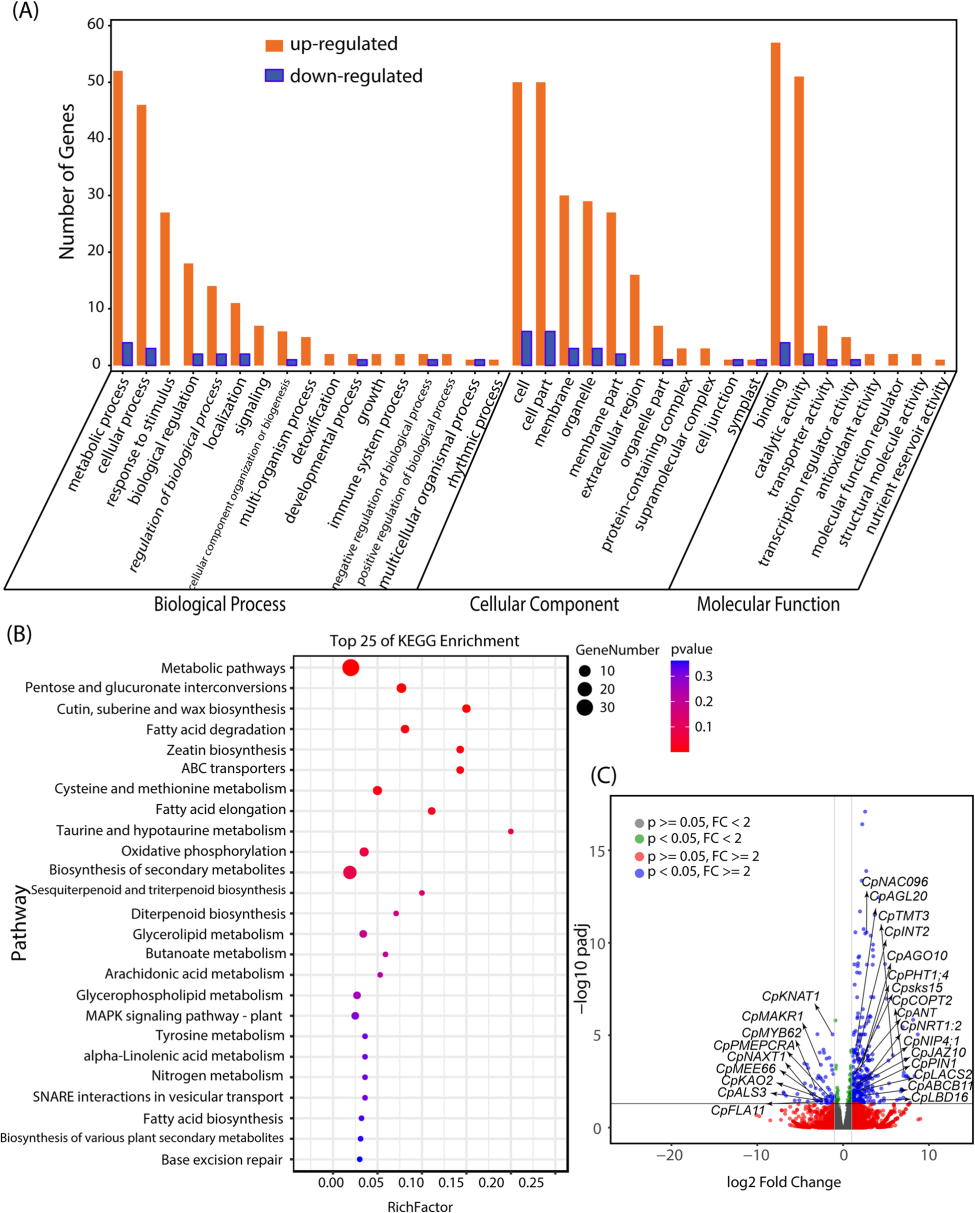
**A** Gene Ontology classification of the DEGs of GA_3_-treated female papaya (*Carica papaya* L. cv. Zhonghuang) roots. **B** Top 25 categories of KEGG enrichment of the DEGs. **C** Volcano plots of the DEGs. p, adjusted *p* value; FC, fold change

To understand the mechanism of how the roots of papaya respond to the GA_3_ treatment, the roots of GA_3_-treated and control papaya were collected for RNA-Seq analysis. A total of 343 differentially expressed genes (DEGs) were identified by DESeq2 analysis with 61 down-regulated and 282 up-regulated DEGs. GO enrichment analysis of the DEGs showed that the greatest distribution of biological processes corresponded to the metabolic process, followed by cellular process, response to stimulus. In the cellular component category, many DEGs were clustered in cell, cell part and organelle categories. In the molecular function category, the top three distributions were binding, catalytic activity and transporter activity (Fig. 2A). In order to further clarify the functions of DEGs, they were subjected to KEGG enrichment. Eight categories were significantly enriched (*p*<0.05) such as metabolic pathways, pentose and glucuronate interconversions, cutin, suberine and wax biosynthesis, fatty acid degradation, zeatin biosynthesis, ABC transporters, cysteine and methionine metabolism, fatty acid elongation (Fig. 2B).

Many DEGs genes encode transporters were identified upregulated in the GA_3_-treated papaya roots such as *CpTMT3* (*TONOPLAST MONOSACCHARIDE TRANSPORTER3*), *CpNRT1:2 (ABA-IMPORTING TRANSPORTER 1:2*), *CpPHT1;4* (*PHOSPHATE TRANSPORTER 1;4*) *CpINT2* (*INOSITOL TRANSPORTER 2*), *CpCOPT2* (*COPPER TRANSPORTER 2*), *CpABCB11* (*ATP-BINDING CASSETTE B11*), *CpNIP4;1* (*NOD26-LIKE INTRINSIC PROTEIN 4;1*). While *CpNAXT1* (*NITRATE EXCRETION TRANSPORTER1*) showed a downregulation in the roots of papaya by GA_3_ treatment (Fig. 2C). The results suggested that GA_3_-treated female papaya had a higher demand for nutrient and water from soil with the higher growth and yield. Papaya also showed the downregulation of *CpALS3* (*ALUMINUM SENSITIVE 3*) and *CpMYB62*, indicating a stronger resistance to the toxic effects of aluminum (Al) and phosphate (Pi) starvation under the condition of GA_3_ treatment.

Additionally, plant hormone associated genes were also differentially expressed in GA_3_-treated papaya roots. For example, *CpKAO2* (*ENT-KAURENOIC ACID HYDROXYLASE 2*), *CpMAKR1* (*MEMBRANE-ASSOCIATED KINASE REGULATOR 1*) and *CpBP* (*BREVIPEDICELLUS 1*) were downregulated, whereas *CpJAZ10* (*JASMONATE-ZIM-DOMAIN PROTEIN 10*), *CpPIN1* (*PIN-FORMED 1*) and *CpLBD16* (*LATERAL ORGAN BOUNDARIES-DOMAIN 16*) were upregulated by GA_3_ application (Fig. 2C).

### Effects of GA3 on metabolic composition in the roots and rhizosphere soils of female papaya

**Fig. 3:**
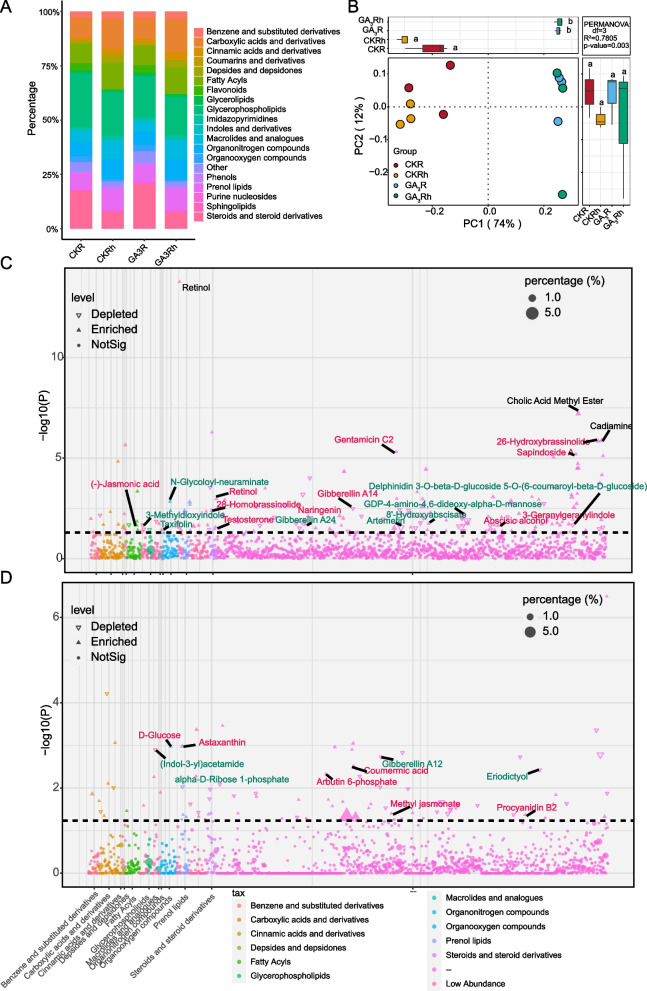
**A** Relative abundance of Top 19 metabolic taxonomy in the samples. **B** PCoA plot showing the metabolic separation in roots and rhizosphere soils of papaya (*Carica papaya* L. cv. Zhonghuang) influenced by GA_3_ application. **C**-**D **Manhattan plot showing metabolites of the roots (C) and rhizosphere soils (**D**) of papaya. Each point represented a metabolite. The size of each point represented the relative abundance of the metabolite, and the colors of the point denoted the metabolite categories. The upward triangles represented that the metabolites were significantly up-regulated by GA_3_ application, while the downward triangles represented significantly down-regulated compared with CK

To explore the effects of GA_3_ on roots and rhizosphere soils, the roots and rhizosphere soils of CK group and GA_3_-treated group were collected for LC-MS/MS non-targeted analyses. A total of 2602 metabolites were detected from all samples based on The Human Metabolome Database (HMDB). The metabolites were annotated to 80 taxonomic categories, including glycerophospholipids (14.8%-25.3%), steroids and steroid derivatives (7.4%-20.5%), carboxylic acids and derivatives (9.6%-15.3%), fatty acyls (9.1%-11.6%), phenol lipids (7.6%-9.9%), organonitrogen compounds (6.3%-11.6%), macrolides and analogues (4.6%-10.3%), flavonoids (0.0%-3.5%), and phenols (0.8%-1.1%) (Fig. 3A).

These taxa in the different compartments were subject to multiple difference comparison analysis, by which the significant differences were observed between the roots and rhizosphere soils of papaya. For example, the relative abundance of steroids and steroid derivatives, organooxygen compounds, flavonoids and indoles and derivatives was significantly higher in roots than in rhizosphere. While cinnamic acids and derivatives showed an opposite trend. On the other hand, when compared to the CK group, the relative abundance of flavonoids was found increased in the roots of GA_3_-treated papaya (Table S1). Principle coordinate analysis (PCoA) revealed a clear separation in the metabolic composition between CK and GA_3_-treat groups (*p*-value <0.01 in Permanova analysis). PC1 and PC2 explained 74% and 12% of the variance in the metabolic composition, respectively (Fig. 3B).

### Differential abundance analysis of metabolites in roots and rhizosphere soils

DESeq2 analysis was employed to identify the differential metabolites in the papaya roots and rhizosphere soils caused by GA_3_ treatment. The results showed that the GA_3_-treated papaya roots had 272 differential metabolites and its rhizosphere soils had 127 differential metabolites. Among the differential metabolites in the roots, 95 metabolites were significantly depleted, such as N-glycoloyl-neuraminate,, GDP-4-amino-4,6-dideoxy-alpha-D-mannose, 8'-hydroxyabscisate, artemetin, 3-methyldioxyindole, gentamicin C2, sapindoside A, taxifolin, norgestrel, glucocerebrosides, auramycinone, 5,6-DHET, validamycin A, hexadecanoic acid and gibberellin A24. In contrast, 172 metabolites were enriched, including 26-hydroxybrassinolide, 28-homobrassinolide, 3-geranylgeranylindole, 2-(alpha-D-Mannosyl)-3-phosphoglycerate, abscisic alcohol, gentamicin C2, delphinidin 3-O-beta-D-glucoside 5-O-(6-coumaroyl-beta-D-glucoside), sapindoside A, taxifolin, testosterone, gibberellin A14, (-)- jasmonic acid, retinol and naringenin (Figure 3C).

Compared to the CK group, the rhizosphere soils of the GA_3_-treated papaya showed fewer differential metabolites with 73 metabolites enriched and 49 metabolites depleted. For example, the abundance of 4-guanidinobutyric acid, violacein, (Indol-3-yl) acetamide, eriodictyol, alpha-D-Ribose 1-phosphate and gibberellin A12 were reduced significantly. While the abundance of astaxanthin, arbutin 6-phosphate, D-glucose, coumermic acid, methyl jasmonate and procyanidin B2 were increased in the rhizosphere soils of GA_3_-treat papaya (Fig. 3D). The abundence of stearoylethanolamide, chenodeoxycholate and isotabtoxin were significantly reduced (*p*<0.05) in the roots and rhizosphere of GA_3_-treated papaya. Wheras, the abundence of ganoderenic acid A and diosgenin glucoside were significantly increased in both niches by application of GA_3_ (*p*<0.05).

### Effects of GA_3_ on microbiota composition in the roots and rhizosphere soils of female papaya

For 16S rRNA sequencing data, a total of 960,275 reads were obtained from all samples. The average GC content of 16S bacterial rRNA was 55.75%, and the bases with mass value greater than or equal to 30 accounted for 96.72% of the total bases. 1,895 operational taxonomic units (OTUs) at 97% identity were found with the number of OTUs ranging from 824 to 1,754 per sample (Table S2A), from the samples derived from the roots and rhizosphere soils of CK and GA_3_-treat papaya. The coverage for the observed OTUs was 99.83 ± 0.01% (mean ± sem).

The ITS amplicon was also sequenced using the DNA from the roots and rhizosphere soils of papaya. A total of 511,030 pairs of reads were obtained from the 12 samples. The average GC content of ITS1 was 45.76% and the bases with mass value greater than or equal to 30 accounted for 99.14% of the total bases. 1,021 OTUs at 97% identity were obtained from the entire sample, with the number of OTUs ranging from 268 to 638 per sample (Table S2B).

**Fig. 4:**
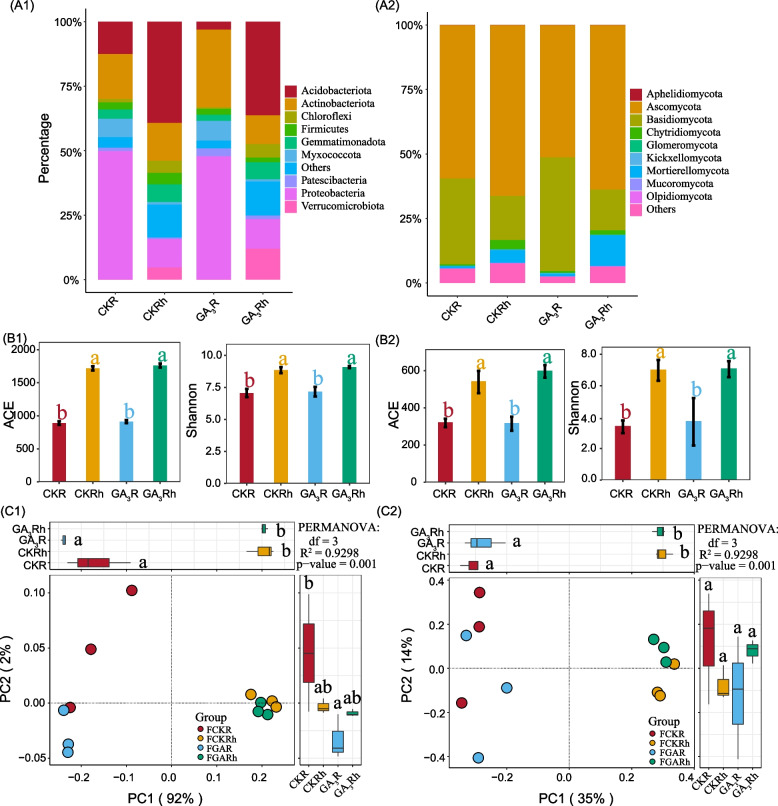
Taxonomic composition of the bacteria (A1) and fungi (A2) at phylum levels in roots and rhizosphere soi of papaya (*Carica papaya* L. cv. Zhonghuang). Alpha diversity analysis showed that the richness (ACE) and diversity (Shannon) of bacteria (B1) and fungi (B2) in roots and rhizosphere soils between CK and GA_3_ treatment. The different letters meant significant difference (*p*<0.05). PCoA analysis demonstrating of bacteria (C1) and fungal (C2) community in roots and rhizosphere soils between CK and GA_3_ treatment. The differential analysis on PC1 and PC2 revealed the differences in microbial composition. Different letters represented significant differences (*p* <0.05). Permanova analysis revealed significant differences between every two microbial community structures (*p* <0.01)

In all samples, the most dominant bacterial phyla were Proteobacteria (11.1%-49.9%), Acidobacteriota (3.1%-39.3%) and Actinobacteriota(11.1%-30.1%), followed by Firmicutes (1.7%-4.4%) and Gemmatimonadota (2.5%-6.9%) (Fig. 4A1). The relative abundance of Acidobacteriota and Chloroflexi was increased in roots and rhizosphere soils of the GA_3_ treatment group. Conversely, that of Proteobacteria and Myxococcota showed opposite results. Additionally, the abundance of Actinobacteriota were also decreased by GA_3_ treatment in the roots of female papaya (Table S3). Ascomycota (51.3%-66.3%) and Basidiomycota (15.8%-44.1%) were the most dominant fungal phyla in the roots and rhizosphere soils, followed by Chytridiomycota (0.5%-3.5%) (Fig. 4A2). The abundance of Chytridiomycota and Mortierellomycota were increased in the roots and rhizosphere soils of GA_3_-treat papaya, respectively (Table S3).

The bacterial and fungal alpha diversity (Shannon index) did not differ between the CK and GA_3_-treat groups, but the ACE and Shannon indexes of roots were lower compare to the rhizosphere soils (Fig. 4B1 and B2). PCoA showed the bacterial and fungal community of roots and rhizosphere soils were well separate along the first axe. And the bacterial community structure of roots was less complex in GA_3_-treat papaya compared to the control papaya (Fig. 4C).

**Fig. 5:**
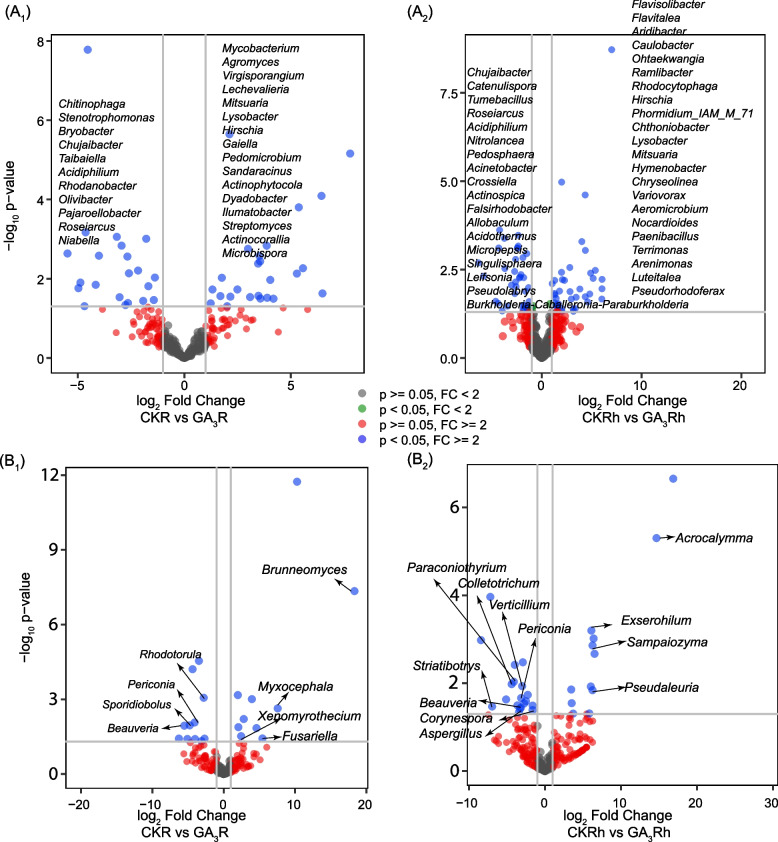
Volcano plots showing differential expressed genera of bacteria (**A**) and fungi (**B**) in roots and rhizosphere soils between CK and GA_3_ treatment. 1, roots; 2, rhizosphere soils. FC means fold change

To predict ecological functions of bacteiral composition in roots and rhizosphere of CK and GA_3_-treated papaya at the taxonomic level, FAPROTAX software was employed for this analysis. Abundant bacterial species in roots were attributed to chemoheterotrophy (33.95-38.32%), aerobic chemoheterotrophy (35.58-37.18%), nitrate reduction (7.54-9.76%), and predatory or exoparasitic (7.10-7.52%). Functions attributed to nitrogen respiration and nitrate respiration showed lower abundance in GA_3_-treated papaya roots (0.43%) compared with control papaya roots (3.67%) (Fig. S1A1). While abundant bacterial species in rhizosphere were attributed to chemoheterotrophy (36.60-37.96%), aerobic chemoheterotrophy (32.16-33.88%), manganese oxidation (5.06-6.00%), fermentation (4.59-5.17%) and nitrate reduction (2.71-4.02%) (Fig. S21A2). Functions of fungal composition in roots and rhizosphere of CK and GA_3_-treated papaya were mainly predicted to pathotroph, symbiotroph and saprotroph by FUNguild software. In roots, functions attributed to pathotroph and saprotroph showed lower, while the abundance of symbiotroph was increased in the GA_3_-treated papaya (Fig. S1B1). This pattern was also shown in rhizosphere (Fig. S1B2).

### Differential abundance analysis of bacterial and fungal genera

The differential abundance of bacteria and fungi in roots and rhizosphere soils were identified by DESeq2.The Differential abundance of bacteria in the roots and rhizosphere soils was 46 and 79 genera (Fig. 5A1and A2), respectively, and the differential abundance of fungi in the roots and rhizosphere soils was 19 and 28 genera (Fig. 5B1and B2), respectively. Compared to the roots of CK papaya, the bacteria genera, including *Chitinophaga*,*Stenotrophomonas*, *Bryobacter*, *Chujaibacter,Taibaiella*, *Acidiphilium*, *Rhodanobacter*, *Olivibacter*, *Pajaroellobacter*, *Roseiarcus*, *Niabella,* were significantly decreased (*p*<0.05) in the GA-treated papaya, while the genera such as *Mycobacterium*, *Agromyces*, *Virgisporangium*, *Lechevalieria*, *Mitsuaria*, *Lysobacter*, *Hirschia*, *Gaiella*, *Pedomicrobium*, *Sandaracinus*, *Actinophytocola*, *Dyadobacter*, *Ilumatobacter*, *Streptomyces*, *Actinocorallia*, and *Microbispora* were significantly enriched (*p*<0.05) (Fig. 5A1).

In comparison with the rhizosphere soils of CK papaya, the counterpart of GA-treated papaya was significantly depleted with some bacteria genera, including *Chujaibacter*, *Catenulispora*, *Tumebacillus, Roseiarcus*, *Acidiphilium*, *Pedosphaera*, *Burkholderia-Caballeronia-Paraburkholderia*, *Nitrolancea*, *Acinetobacter*, *Crossiella*, *Actinospica*, *Falsirhodobacter*, *Allobaculum*, *Acidothermus*, *Micropepsis*, *Singulisphaera*, *Leifsonia* and *Pseudolabrys*, but significantly enriched with other genera, including *Flavisolibacter*, *Flavitalea*, *Aridibacter*, *Caulobacter*, *Ohtaekwangia*, *Ramlibacter*, *Rhodocytophaga*, *Hirschia*, *Phormidium_IAM_M_71*, *Chthoniobacter*, *Lysobacter*, *Mitsuaria*, *Hymenobacter*, *Chryseolinea*, *Variovorax*, *Aeromicrobium*, *Nocardioides*, *Paenibacillus*, *Terrimonas*, *Arenimonas*, *Luteitalea*, and *Pseudorhodoferax* (Fig. 5A2).

For fungi, the roots of GA_3_-treat papaya significantly enriched in genera *Rhodotorula*, *Periconia*, *Sporidiobolus*, *Beauveria*, but depleted in genera *Brunneomyces*, *Myxocephala*, *Xenomyrothecium* and *Fusariella* (Fig. 5B1). In the rhizosphere soils of GA_3_-treated papaya, the abundance of genera *Paraconiothyrium*, *Colletotrichum*, *Verticillium*, *Periconia*, *Striatibotrys*, *Beauveria*, *Corynespora* and *Aspergillus* was significantly decreased, while that of *Acrocalymma*, *Exserohilum*, *Sampaiozyma* and *Pseudaleuria* was significantly increased (Fig. 5B2).

## Discussion

Root is the key organ for the uptake of water and nutrient from soil, which directly affect plant growth, development, yield and stress tolerance [12, 16]. It closely associated with endophytic and rhizosphere microorganisms, forming a dynamically balanced ecosystem to improve plant growth and resistance [17–20]. It has been reported that exogenous application of GA_3_ had positive effects on plant height, peduncle length and flower number on female papaya [9]. However, the responses of roots and rhizosphere to exogenous GA_3_ were not yet understood. In the present study, the aboveground phenotypes of GA_3_-treated papaya are consistent with the previous study. For belowground phenotype, exogenous GA_3_ enhanced the lateral root growth of female papaya so that the plants had the physical advantages of water and nutrient uptake.

In the transcription profile, exogenous GA_3_ affected 343 genes differentially expressed in female papaya roots. GO enrichment and KEGG enrichment analysis showed that the DEGs were significantly enriched in transporter activity and ABC transporters category, respectively. Among the DEGs, various genes encoding transporters were upregulated such as *CpTMT3*, *CpNRT1:2*, *CpPHT1;4*, *CpINT2*, *CpCOPT2*, *CpABCB11*, *CpNIP4;1* [21–25]. While *CpNAXT1* showed downregulation in the roots of papaya by GA_3_ treatment. The results demonstrated that GA_3_-treated female papaya root expressed more correspond transporters and downregulated excretion transporters to absorb water and various nutrients including sugar, phosphate, ABA and metal ion from soil. In addition, GA_3_-treated papaya also showed downregulation of *CpALS3* and *CpMYB62*, indicating a stronger resistance to aluminum and phosphate starvation [26, 27].

The plant hormone associated-genes also differentially expressed in GA_3_-treated female papaya roots. *CpKAO2* that catalyzes the conversion of ent-kaurenoic acid to GA_12_, the precursor of all GAs [28], was downregulated in GA_3_ treated papaya roots. Moreover, GA_24_ and GA_12_ were depleted in the roots and rhizosphere soils of GA_3_-treated papaya respectively. The results suggested a negative feedback regulation of GA biosynthesis in the GA-treated papaya roots under the condition of GA_3_ application.

Auxin-related genes also occurred in the DEGs, such as *CpPIN1*, *CpLBD16* and *CpBP*. Auxin (indole-3-acetic acid [IAA]) is an important plant hormone for regulation of lateral root development [29]. It is mostly produced in the shoot apices and is actively transported basipetally in the polar auxin transport stream, involving basally localized PIN proteins [29, 30]. It has reported that spraying GA3 increased auxin levels as much as 8-fold and decreased indole-3-acetyl aspartic acid levels in dwarf pea (*Pisium sativum* cv. Little Marvel) [31]. *Arabidopsis* mutants deficient in GA biosynthesis and signaling showed a reduction of auxin transport and PIN protein levels in the inflorescences [32]. Moreover, auxin efflux regulated by PIN proteins was required for lateral root formation [33]. *LBD16* encodes plant-specific transcription factors, which acts downstream of the *AUXIN1* and *LIKE-AUXIN3* auxin influx carriers to control lateral root initiation and development [33–35]. *BP* overexpression in *Arabidopsis* caused reductions in the length and number of lateral roots through inhibiting IAA-induced lateral root growth [36, 37]. In GA_3_-treated papaya root, *CpPIN1* and *CpLBD16* were upregulated, while the expression of *CpBP* was repressed. The results suggested that treatment of female papaya with GA_3_ resulted in the promotion of lateral root formation and development by upregulating *CpLBD16* and downregulating *CpBP*.

BRs and JAs mediate plant growth, development and defense responses [38, 39]. 26-hydroxybrassinolide and 28-homobrassinolide were enriched in GA_3_-treated papaya roots. *CpMAKR1* that involved in brassinosteroid and receptor-like kinase signaling, was downregulated in GA_3_-treated papaya roots. Jasmonic acid and methyl jasmonate were also enriched in the roots and rhizosphere of GA_3_-treated papaya. *CpJAZ10* whose *Arabidopsis* ortholog encodes one of the jasmonate ZIM domain (JAZ) transcriptional repressor proteins [40], showed an upregulation in GA_3_-treated papaya roots. These findings suggested that GA_3_-treated papaya roots exhibited feedback control of brassinolide and jasmonate signaling in root development and defense.

Endophyte colonizer inside plants can benefit their host plants directly through improving plant nutrient uptake and by modulating growth and stress related phytohormones, whose diversity depends on plant and environment specific factors [41]. In the GA_3_-treated female papaya roots, bacterial functions attributed to nitrogen respiration and nitrate respiration showed higher abundance. Moreover, the species of *Candidatus solibacter* and *Bryobacter* have been reported to increase in abundance under the condition of appropriate nitrate ammonium ratio [42]. They had reduced abundance in the GA_3_-treated papaya roots. *Candidatus solibacter* is a moderately acidophilic and multifunctional heterotrophic bacterium, with the ability to reduce nitrate and nitrite [43]. The results collectively indicated that GA_3_-treated female papaya had less demands for nitrate in comparison with control papaya, leading to the decrease of *Candidatus solibacter* and *Bryobacter* in abundance. Though the two beneficial genera were decreased by GA_3_ treatment, other beneficial genera showed relatively higher abundance like *Mycobacterium*, *Mitsuaria*, and *Actinophytocola*.

There were also fungal composition alterations in roots and rhizosphere of GA-treated papaya. After GA_3_ treatment, the fungi with functions of pathotroph and saprotroph showed lower abundance, while the abundance of symbiotroph was increased in papaya roots and rhizosphere. Especially, the pathogenic species *Colletotrichum* and *Verticillium*, which caused anthracnose [44] and verticillium wilt [45], respectively, were decreased in abundance in the rhizosphere of GA_3_-treated papaya.

## Conclusions

**Fig. 6:**
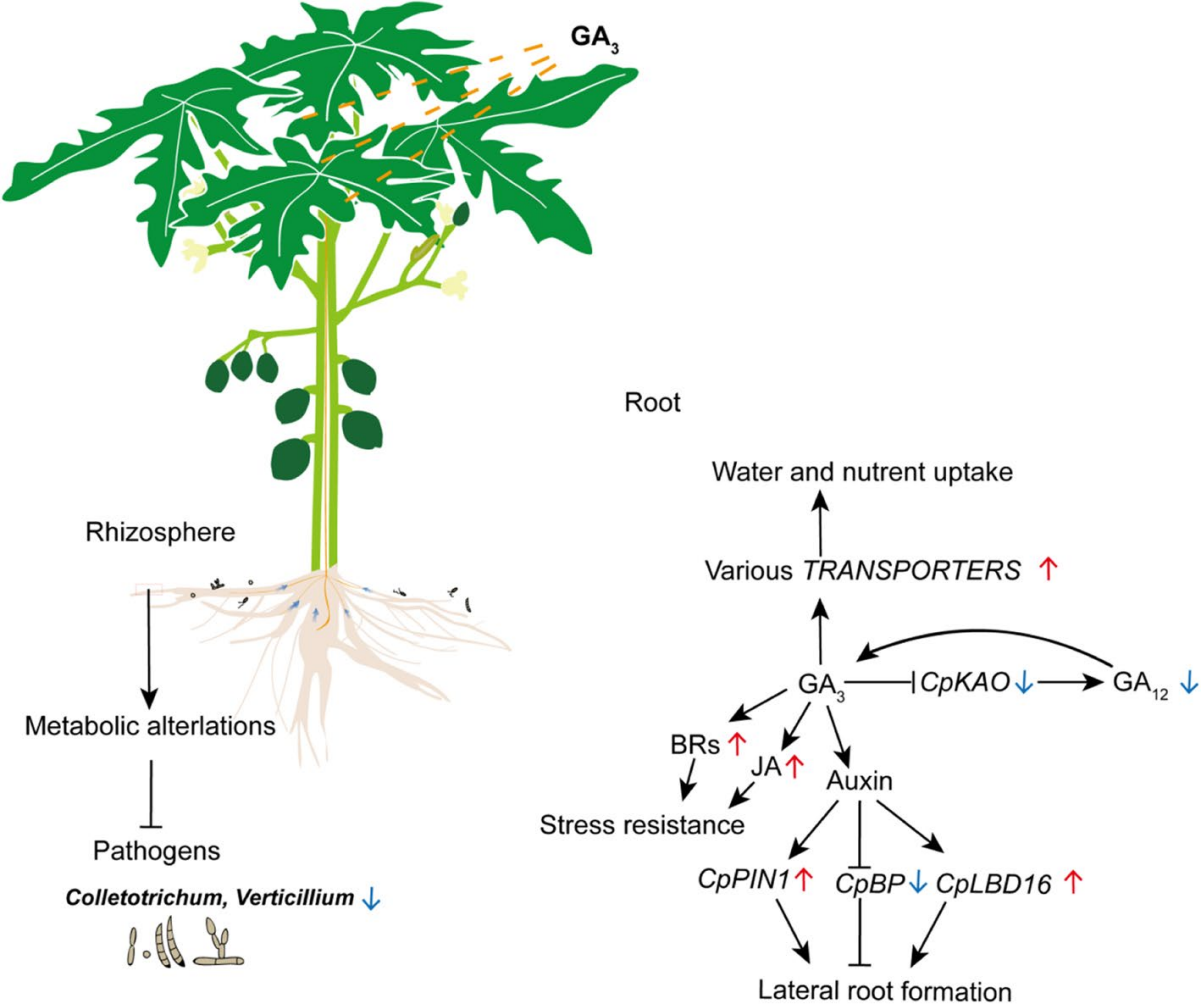
Conclusion of responses of roots and rhizosphere to GA_3_ application on papaya(*Carica papaya* L. cv. Zhonghuang) shoot apicles. Red arrows indicated upregulation and blue arrows indicated downregulation

Exogenous GA_3_ might increase the level of auxin, which was transported to roots by CpPIN1, where auxin upregulated *CpLBD16* and repressed *CpBP* to promote the lateral root initiation and development. Additionally, papaya’s corresponding transporters (*CpTMT3*, *CpNRT1:2*, *CpPHT1;4*, *CpINT2*, *CpCOPT2*, *CpABCB11*, *CpNIP4;1*) were upregulated and excretion transporter *CpNAXT1* was downregulated for water and nutrients uptake with exogenous GA_3_ application. Moreover, in GA_3_-treated papaya roots, *CpALS3* and *CpMYB62* were downregulated, suggesting a stronger abiotic resistance to aluminum toxic and phosphate starvation. On the other hand, BRs and JAs were enriched in the roots and rhizosphere of GA_3_-treated papayas. The upregulation of the two hormones might result in the reduction of pathogens in roots and rhizosphere such as *Colletotrichum* and *Verticillium*. GA_3_-treated female papaya increased the abundance of beneficial bacteria species including *Mycobacterium*, *Mitsuaria*, and *Actinophytocola*, but decreased that of the genera *Candidatus* and *Bryobacter* for that it required less nitrate. Therefore, the roots and rhizosphere of female papaya positively respond to exogenous GA_3_ to promote its development and stress tolerance (Fig. 6).

## Materials and methods

### Sampling and preparation of roots and rhizosphere soils

The cultivar of papaya used in the experiments was Zhonghuang. The materials were planted in filed at experimental base of Fujian Agriculture and Forestry University (25°13′15″N, 117°41′55″E), Wufeng Town, Yongchun County, Quanzhou City, Fujian Province, China.The female papayas were treated with a GA solution at 150 μM on shoot apical meristem weekly. After the eighth treatment, the roots and rhizosphere soils of CK and GA_3_-treated papaya were collected. Each sample consist of three replicates. The roots were soon washed using sterilized ddH_2_O. We stored about 2 g of each sample at -80 ºC until used in the RNA extraction. The left roots were then surface-sterilized with 100% ethanol for 1 min, 2.5% fresh bleach for 30 min and 100% ethanol for 1 min. Afterwards, the roots were also stored at -80 ºC before metabolites and total DNA extraction. The rhizosphere soils of each sample was mixed well and removed visible roots and stones before stored at -80 ºC. Our experimental research and field studies on (*Carica papaya* L. cv. Zhonghuang), including the collection of plant material, complied with all relevant institutional, national, and international guidelines and legislation.

### Morphological analysis of papaya roots

The root biomass, total length and root surface area of CK and GA_3_-treated papaya were measured with an Epson Expression 12000XL instrument by scanning the root plane image in greyscale at 300 dpi, followed by WinRHIZO root system analysis as described previously [46]. Various orders of lateral roots of CK and GA_3_-treated papaya were classified according the diameter of roots. The difference of total root lenth of different orders of lateral roots were analyzed by Student's t-test.

### Transcriptome analysis

The total RNA was extracted from roots of each papaya. The RNA purity and quantity were assessed by the NanoDrop 2000 spectrophotometer and agarose gel. The RNA was reverse-transcribed into mRNA for PCR reaction using oligo primers. The PCR products were purified before sequencing on an Illumina MiSeq platform. The raw reads were filtered using Trimmomatic software to obtain clean reads [47]. The clean reads were aligned to the papaya reference genome using Hisat2 [48]. The mapped reads of each sample were assembled by StringTie [48].

The software FeatureCounts was used to count the number of reads mapped to each gene, and the fragments per kilobase of transcript per million mapped reads (FPKM) of each gene were calculated based on the length of the gene and the read count mapped to this gene. We defined DEGs with an adjusted *p*-value < 0.05 and fold change value >2 by DESeq2 software package in the R [49]. The DEGs were subjected to functional enrichment analysis with Gene Ontology (GO) terms, and Kyoto Encyclopedia of Genes and Genomes (KEGG) pathway categories [50] by online analysis softwares in Omicshare (https://www.omicshare.com/). The GO terms and KEGG pathways with a *p*-value of <0.05 were considered to be significantly enriched.

### Metabolites extraction and LC-MS/MS analysis

LC-MS/MS analyses were performed to identify metabolites across samples using an UHPLC system (1290, Agilent Technologies) with a UPLC BEH Amide column (1.7 μM 2.1*100 mm, Waters) coupled to TripleTOF 5600 (Q-TOF, AB Sciex). Then 100 μg of each sample was extracted with 300 μL of methanol, adding 20 μL internal standard substances with vortex for 30 s, and subsequently ultrasound treated for 10min (incubated in ice water) and incubation for 1h at -20 °C to precipitate proteins. After centrifugation at 13,000 rpm for 15 minutes at 4 °C, the supernatant was transferred into a fresh 2 mL LC/MS glass vial. 20 μL supernatant from each sample was pooled and 200 μL mixed supernatant was taken for the UHPLC-QTOF-MS analysis.

The output data were converted to the mzXML format using ProteoWizard, and processed by R package XCMS (version 3.2). The preprocessing results generated a data matrix that consisted of the retention time (RT), massto-charge ratio (m/z) values, and peak intensity. The CAMERA package in R was used for peak annotation after XCMS data processing. In-house MS2 database was applied in metabolites identification.

### 16S rRNA and ITS amplicon sequencing

Total genomic DNA was extracted using the Fast DNATM Spin Kit (MP Biomedicals, LLC, Santa Ana, CA, United States). The DNA purity and quantity were assessed by the NanoDrop 2000 spectrophotometer (Thermo Fisher) and agarose gel. Using the genomic DNA as template, the hypervariable V3-V4 regions of 16S rRNA gene were amplified by PCR with the primers 341F and 785R [51]. The fungal ITS1 regions of ITS were amplified by PCR with the primers ITS5-1737F and ITS2-2043R [52]. The amplification products were collected from a 2% agarose gel and purified by Vazyme VAHTSTM DNA Clean Beads. The sequencing libraries were established using TruSeq Nano DNA LT Library Prep Kit (Illumina, SD, USA) and then sequenced on an Illumina MiSeq platform (Illumina, SD, USA).

The Illumina paired-end raw data were filtered using Trimmomatic [47], then the primer sequences were identified and removed using Cutadapt (Martin, 2011) followed by paired-end pairing using USEARCH (Edgar, 2013). The chimera readings were detected and removed by UCHIME to obtain high-quality sequences for analysis [53]. Sequences with 97% similarity were clustered at the sequence level using USEARCH with a default threshold of 0.005% of all sequences to filter operational taxonomic units (OTUs). And the Ribosomal Database Project (RDP) classifier was used to annotate the species of all representative reads with confidence threshold 70% according to the Silva database [54].

## Statistical analysis

Shannon index, and richness index (ACE ) estimator were used to analyze the alpha diversity by the phyloseq package [55]. Principal Component Analysis (PCoA) was performed using Bray-Curtis algorithm of Quantitative Insights into Microbial Ecology (QIIME) and R software to compare the similarity of species diversity in different samples [56]. Permutational Multivariate Analysis of Variance (PERMANOVA) and paired PERMANOVA using vegan package at 999 permutations and α = 0.05 to test metabolites dissimilarities [57]. The metabolites and microbial genera significantly depleted or enriched were determined by DESeq analysis. Manhattan plot and volcano plot were employed using the R language to illustrate the differential metabolites and differential microbes of the roots and rhizoshpare.

## Supplementary Information

**Additional file 1** .

**Additional file 2:** Table S1. Comparison of relative abundance of metabolites in each group. Table S2A Bacterial 16S rRNA Sequencing Data Quality Assessment. Table S2B Fungi ITS1 Sequencing Data Quality Assessment. Table S3. Comparison of relative abundance of bacteria and fungi in each group. Figure S1 Functions of bacterial (A) and fungal (B) composition in roots and rhizosphere of female papaya. 1, roots; 2, rhizosphere soil.

## Data Availability

All data generated or analysed during this study are included in this published article and its supplementary information files. The RNAseq and 16s/ITS amplicon sequencing data has been deposited in SRA (Sequence ReadArchive). It is accessible by searching the BioProject ID: PRJNA857499 in NCBI.
