# Supplementary material for: Responses of roots and rhizosphere of female papaya to the exogenous application of GA3
Source: BMC Plant Biol. 2023 Jan 16;23:35. doi: 10.1186/s12870-022-04025-6 (PMC9841646; doi:10.1186/s12870-022-04025-6)

Table S1. Comparison of relative abundance of metabolites in each group.

|  | FCKR | FGA_3_R | FCKRh | FGA_3_Rh |
| --- | --- | --- | --- | --- |
| Glycerophospholipids | 25.3 a | 14.8 a | 20.3 a | 15.7 a |
| Steroids and steroid derivatives | **17 a** | **20.5 a** | 7.4 b | 7.5 b |
| Carboxylic acids and derivatives | 9.6 ab | 10.4 b | 14.1 ab | **15.3 a** |
| Fatty Acyls | 9.1 ab | 9.9 b | 12.5 a | 11.6 ab |
| Prenol lipids | 7.6 a | 9 a | 9.7 a | 9.9 a |
| Organonitrogen compounds | 6.9 a | 6.3 a | 9.5 a | 11.6 a |
| Macrolides and analogues | 4.6 a | 5.8 a | 8.4 a | 10.3 a |
| Benzene and substituted derivatives | 2.6 a | 2.8 a | 3 a | 3.4 a |
| Cinnamic acids and derivatives | 1.1 b | 1.6 b | **3.1 a** | **2.9 a** |
| Depsides and depsidones | 0.9 ab | 0.5 b | 3.5 a | **3.6 a** |
| Organooxygen compounds | **2.3 a** | **3 a** | 0.8 b | 0.9 b |
| Flavonoids | **3.5 a** | **2.5 b** | 0 c | 0 c |
| Imidazopyrimidines | 1.1 a | 1.3 a | 2 a | 2.3 a |
| Glycerolipids | 0.9 a | 1.6 a | 1 a | 0.6 a |
| Phenols | 0.8 a | 0.9 a | 1.1 a | 0.8 a |
| Purine nucleosides | 0.5 a | 0.8 a | 0.6 a | 0.6 a |
| Sphingolipids | 0.5 a | 0.5 a | 0.6 a | 0.6 a |
| Coumarins and derivatives | 0.5 ab | **1.1 a** | 0 b | 0 b |
| Indoles and derivatives | **0.7 a** | **0.7 a** | 0 b | 0 b |
| Others | 4.4 a | 5.8 a | 2.4 b | 2.3 b |

Note: The lowercase letters above the bars mean significant difference (P<0.05)

Table S2A Bacterial 16S rRNA Sequencing Data Quality Assessment

| Sample ID | Raw Reads | Clean Reads | Effective Reads | AvgLen(bp) | GC(%) | Q20(%) | Q30(%) | Effective(%) | OTU_Num | Coverage |
| --- | --- | --- | --- | --- | --- | --- | --- | --- | --- | --- |
| FCKR01 | 79866 | 79577 | 65999 | 402 | 54.66 | 99.31 | 96.99 | 82.64 | 824 | 0.9985 |
| FCKR02 | 79849 | 79546 | 69421 | 402 | 54.86 | 99.31 | 97 | 86.94 | 879 | 0.9987 |
| FCKR03 | 80242 | 79978 | 67377 | 401 | 53.96 | 99.31 | 96.96 | 83.97 | 852 | 0.9983 |
| FCKRh01 | 79494 | 79196 | 76161 | 416 | 57.22 | 99.12 | 96.38 | 95.81 | 1612 | 0.9975 |
| FCKRh02 | 80040 | 79727 | 77684 | 417 | 57.21 | 99.12 | 96.37 | 97.06 | 1662 | 0.9979 |
| FCKRh03 | 80280 | 80026 | 78390 | 418 | 57.3 | 99.11 | 96.34 | 97.65 | 1690 | 0.998 |
| FGA_3_R01 | 80206 | 79936 | 69005 | 401 | 54.25 | 99.32 | 96.99 | 86.03 | 890 | 0.9986 |
| FGA_3_R02 | 79575 | 79303 | 67984 | 403 | 54.66 | 99.31 | 96.98 | 85.43 | 910 | 0.9991 |
| FGA_3_R03 | 80721 | 80543 | 64348 | 400 | 55.25 | 99.52 | 97.68 | 79.72 | 849 | 0.9984 |
| FGA_3_Rh01 | 79956 | 79673 | 77638 | 418 | 56.8 | 99.12 | 96.36 | 97.1 | 1754 | 0.9982 |
| FGA_3_Rh02 | 80035 | 79736 | 78124 | 418 | 56.49 | 99.09 | 96.28 | 97.61 | 1726 | 0.9988 |
| FGA_3_Rh03 | 80011 | 79709 | 77942 | 416 | 56.28 | 99.11 | 96.33 | 97.41 | 1681 | 0.9978 |
| Sum/ave | 960275 | 956950 | 870073 | 409 | 55.75 | 99.23 | 96.72 | 90.61 | 1895 | 0.9983 |

Table S2B Fungi ITS1 Sequencing Data Quality Assessment.

| Sample ID | Raw Reads | Clean Reads | Effective Reads | AvgLen(bp) | GC(%) | Q20(%) | Q30(%) | Effective(%) | OTU_Num | Coverage |
| --- | --- | --- | --- | --- | --- | --- | --- | --- | --- | --- |
| FCKR01 | 54094 | 53834 | 53604 | 299 | 40.85 | 99.74 | 98.34 | 99.09 | 294 | 0.9995 |
| FCKR02 | 51183 | 50930 | 50705 | 231 | 47.48 | 99.96 | 99.67 | 99.07 | 320 | 0.999 |
| FCKR03 | 62096 | 61796 | 61626 | 278 | 48.87 | 99.68 | 98.42 | 99.24 | 303 | 0.9997 |
| FCKRh01 | 27594 | 27432 | 27080 | 243 | 45.29 | 99.85 | 99.12 | 98.14 | 503 | 0.9967 |
| FCKRh02 | 33624 | 33414 | 33069 | 249 | 45.82 | 99.84 | 99.06 | 98.35 | 557 | 0.9991 |
| FCKRh03 | 26284 | 26121 | 25765 | 236 | 46.09 | 99.89 | 99.32 | 98.03 | 420 | 0.9976 |
| FGA_3_R01 | 26948 | 26835 | 26711 | 236 | 47 | 99.94 | 99.6 | 99.12 | 255 | 0.9986 |
| FGA_3_R02 | 57052 | 56702 | 56476 | 255 | 44.58 | 99.89 | 99.29 | 98.99 | 308 | 0.9994 |
| FGA_3_R03 | 44673 | 44454 | 44275 | 248 | 48.68 | 99.89 | 99.34 | 99.11 | 332 | 0.9994 |
| FGA_3_Rh01 | 42269 | 42022 | 41633 | 246 | 45.76 | 99.86 | 99.15 | 98.5 | 569 | 0.9996 |
| FGA_3_Rh02 | 43639 | 43270 | 42723 | 246 | 47.98 | 99.86 | 99.15 | 97.9 | 569 | 0.9992 |
| FGA_3_Rh03 | 41574 | 41271 | 40841 | 248 | 45.91 | 99.84 | 99.06 | 98.24 | 629 | 0.9994 |
| Sum/ave | 511030 | 508081 | 504508 | 251 | 46.19 | 99.85 | 99.13 | 98.65 | 1021 | 0.9989 |

Table S3. Comparison of relative abundance of bacteria and fungi in each group

|  |  | FCKR | FGA_3_R | FCKRh | FGA_3_Rh |
| --- | --- | --- | --- | --- | --- |
| Bacteria | Proteobacteria | **49.9 a** | 11.1 b | **47.9 a** | 11.5 b |
| Bacteria | Acidobacteriota | 12.6 b | **39.3 a** | 3.1 b | **36.3 a** |
| Bacteria | Actinobacteriota | 17.4 ab | 14.7 ab | **30.1 a** | 11.1 b |
| Bacteria | Firmicutes | 2.6 a | 4.4 a | 2.3 a | 1.7 a |
| Bacteria | Gemmatimonadota | 3.8 a | 6.9 a | 2.5 a | 6.7 a |
| Bacteria | Verrucomicrobiota | 0 b | 4.7 b | 0 b | 12 a |
| Bacteria | Myxococcota | **7 a** | 0.8 b | **7.5 a** | 0.8 b |
| Bacteria | Chloroflexi | 1.4 b | **4.7 a** | 0.5 b | **5.2 a** |
| Bacteria | Patescibacteria | 1.2 a | 0.5 a | 3 a | 1.3 a |
| Bacteria | Others | 4.2 b | 12.9 a | 3.1 b | 13.3 a |
| Fungi | Aphelidiomycota | 0 a | 0 a | 0 a | 0 a |
| Fungi | Ascomycota | 59.5 a | 66.3 a | 51.3 a | 63.8 a |
| Fungi | Basidiomycota | 33.3 a | 17.1 a | 44.1 a | 15.8 a |
| Fungi | Chytridiomycota | 0.5 b | **3.5 a** | 0.7 b | 1.7 b |
| Fungi | Glomeromycota | 0.1 a | 0.2 a | 0.1 a | 0.1 a |
| Fungi | Kickxellomycota | 0.1 a | 0.1 a | 0.1 a | 0.1 a |
| Fungi | Mortierellomycota | 1 b | 5 ab | 1.2 b | **12.1 a** |
| Fungi | Mucoromycota | 0 a | 0.1 a | 0 a | 0.1 a |
| Fungi | Olpidiomycota | 0 a | 0 a | 0 a | 0 a |
| Fungi | Others | 5.6 a | 7.7 a | 2.5 a | 6.4 a |

The lowercase letters above the bars mean significant difference (P<0.05)

Figure S1 Functions of bacterial (A) and fungal (B) composition in roots and rhizosphere of female papaya. 1, roots; 2, rhizosphere soil.


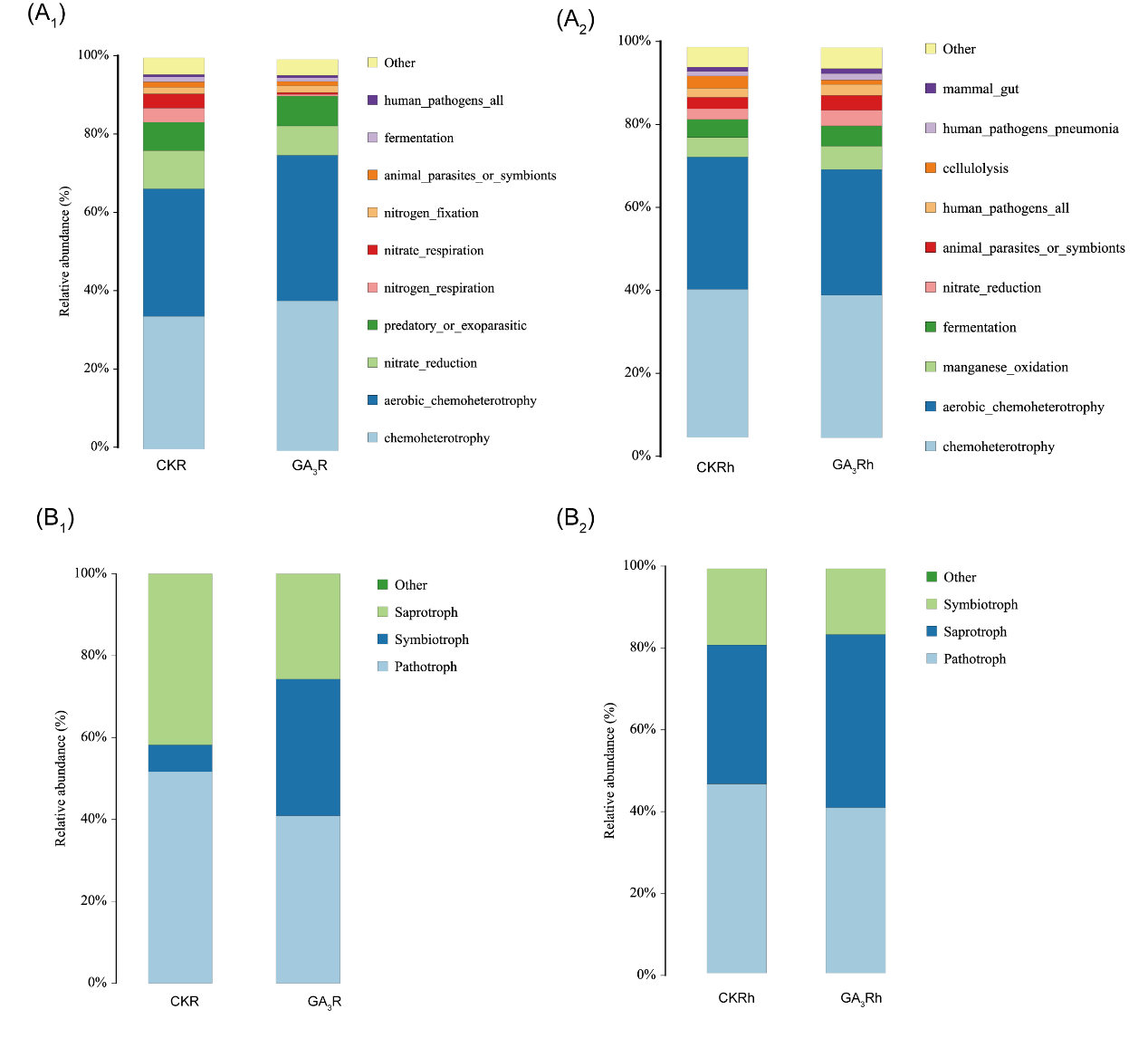

Supplement: Supplementary file 2 — Additional file 2: Table S1. Comparison of relative abundance of metabolites in each group. Table S2A Bacterial 16S rRNA Sequencing Data Quality Assessment. Table S2B Fungi ITS1 Sequencing Data Quality Assessment. Table S3. Comparison of relative abundance of bacteria and fungi in each group. Figure S1 Functions of bacterial (A) and fungal (B) composition in roots and rhizosphere of female papaya. 1, roots; 2, rhizosphere soil. [file 12870_2022_4025_MOESM2_ESM.docx]
